# Three-dimensional morphology of the biramous appendages in *Isoxys* from the early Cambrian of South China, and its implications for early euarthropod evolution

**DOI:** 10.1098/rspb.2023.0335

**Published:** 2023-04-26

**Authors:** Caixia Zhang, Yu Liu, Javier Ortega-Hernández, Joanna M. Wolfe, Changfei Jin, Huijuan Mai, Xianguang Hou, Jin Guo, Dayou Zhai

**Affiliations:** ^1^ Yunnan Key Laboratory for Palaeobiology, Institute of Palaeontology, Yunnan University, Kunming 650091, People's Republic of China; ^2^ MEC International Joint Laboratory for Palaeobiology and Palaeoenvironment, Yunnan University, Waihuan South Road, Chenggong District, Kunming 650500, People's Republic of China; ^3^ Management Committee of the Chengjiang Fossil Site World Heritage, Chengjiang 652599, People's Republic of China; ^4^ Museum of Comparative Zoology and Department of Organismic and Evolutionary Biology, Harvard University, 26 Oxford Street, Cambridge, MA 02138, USA

**Keywords:** early Cambrian Chengjiang biota, early Euarthropod evolution, appendage differentiation, arthrodization, *Isoxys*, micro-computed tomography

## Abstract

Early euarthropod evolution involved a major transition from lobopodian-like taxa to organisms featuring a segmented, well-sclerotized trunk (arthrodization) and limbs (arthropodization). However, the precise origin of a completely arthrodized trunk and arthropodized ventral biramous appendages remain controversial, as well as the early onset of anterior–posterior limb differentiation in stem-group euarthropods. New fossil material and micro-computed tomography inform the detailed morphology of the arthropodized biramous appendages in the carapace-bearing euarthropod *Isoxys curvirostratus* from the early Cambrian Chengjiang biota. In addition to well-developed grasping frontal appendages, *I. curvirostratus* possesses two batches of morphologically and functionally distinct biramous limbs. The first batch consists of four pairs of short cephalic appendages with robust endites with a feeding function, whereas the second batch has more elongate trunk appendages for locomotion. Critically, our new material shows that the trunk of *I. curvirostratus* was not arthrodized. The results of our phylogenetic analyses recover isoxyids as some of the earliest branching sclerotized euarthropods, and strengthens the hypothesis that arthropodized biramous appendages evolved before full body arthrodization.

## 1. Introduction

The presence of segmented appendages with jointed podomeres consisting of substantially hardened (sclerotized) cuticle connected by flexible membranes—formally known as arthropodization—represents the most recognizable character of most extant and extinct euarthropods [1–8]. Arthropodized limbs are enormously plastic in their shape and function [9], and thus represent an important evolutionary innovation that contributes towards the substantial diversity and ecological versatility that characterizes this phylum. Despite the significance of arthropodization as a synapomorphy of Euarthropoda, its precise origin among stem-lineage representatives has proven challenging. The phylogenetically earliest evidence of arthropodization is found among radiodonts, diverse nektobenthic stem-group euarthropods that played an important ecological role in early marine ecosystems during the early Phanerozoic [1,10–12]. Radiodonts possess a single pair of multiarticulated and arthropodized raptorial frontal appendages that mainly served a feeding function, either for grasping, crushing, filter feeding or sediment sifting. Although some radiodonts also feature robust appendicular ‘gnathobase-like structures' associated with the functional head region [12], the rest of the body consists of an unarthrodized trunk with metamerically arranged lateral body flaps for swimming [10]. By contrast, the early evolution of fully arthropodized ventral biramous appendages remains incompletely understood. Suggested evidence for arthropodized legs in Cambrian lobopodians [13] has been regarded as preservation artefacts caused by folding of the flexible or partially decayed cuticle [14]. Since the development of fully arthropodized biramous trunk appendages represents one of the major transitions in early euarthropod evolution [1,2,5,6,9,15–17], resolving this issue carries direct implications for understanding the phylogenetic relationships among early representatives, as well as the emergence of one of the most versatile animal body plans during the Cambrian Explosion.

Recent studies suggest that some of the earliest branching euarthropods bore a broad carapace that covered a weakly sclerotized trunk, and largely homonomous pairs of biramous appendages [1,4,6,17–19]. This view has been reinformed by the recent description of *Erratus sperare* from the early Cambrian Chengjiang biota, which features weakly sclerotized biramous appendages that illustrate the earliest stages of this critical character for euarthropod evolution [20]. Although the biramous appendages of *Erratus* likely illustrate an early stage of arthropodization, this taxon is only known from two specimens that do not preserve the frontal appendages nor a clear dorsal view of the trunk region, which obscures the possible morphological parallels with radiodonts. Among the paraphyletic grade of Cambrian carapace-bearing euarthropods, the isoxyids have been repeatedly compared with radiodonts based on the presence of a pair of raptorial frontal appendages [4,7,17,19], and also the organization of the telson and its adjacent structures [18]. Despite exceptional soft-tissue preservation in isoxyids including the stalked eyes and paired gut diverticulae, the detailed morphology of their body and biramous appendages remains poorly understood, generally obscured by the dorsal carapace covering the entire body [17,18,21–25]. *Surusicaris* from the mid-Cambrian Burgess Shale has some of the most complete biramous trunk appendages in isoxyids described to date [17], interpreted as weakly sclerotized and simple, annulated limbs with an elongate exopod bearing marginal setae. The trunk appendages of *Isoxys volucris* from the early Cambrian Sirius Passet [22] show crudely preserved paddle-shaped exopods, and endopods without clear signs of segmentation. Although the presence of podomere boundaries has been shown in some of the endopods of *I. curvirostratus* and *I. auritus* from Chengjiang [23,24], finer details of the appendicular morphology and functional differentiation are missing in all cases to fully assess their evolutionary and ecological significance. Furthermore, whether the trunk of isoxyids was fully arthrodized or not remains unresolved, although it has been assumed that it might be at least weakly sclerotized [18,21].

Here, we redescribe the morphology of *Isoxys* from the early Cambrian (Stage 3) Chengjiang biota in South China. We employ micro-computed tomography (micro-CT) imaging and three-dimensional computer rendering techniques to investigate the exceptionally preserved pyritized three-dimensional organization of *Isoxys* biramous appendages. We demonstrate that isoxyids had higher degrees of morphological and functional specialization than previously considered [17,18,21–25], and explore their implications for reconstructing the early evolution of the archetypical euarthropod body plan as expressed in members of Deuteropoda [1–6].

## 2. Results

### (a) Preservation

**Figure 1.**
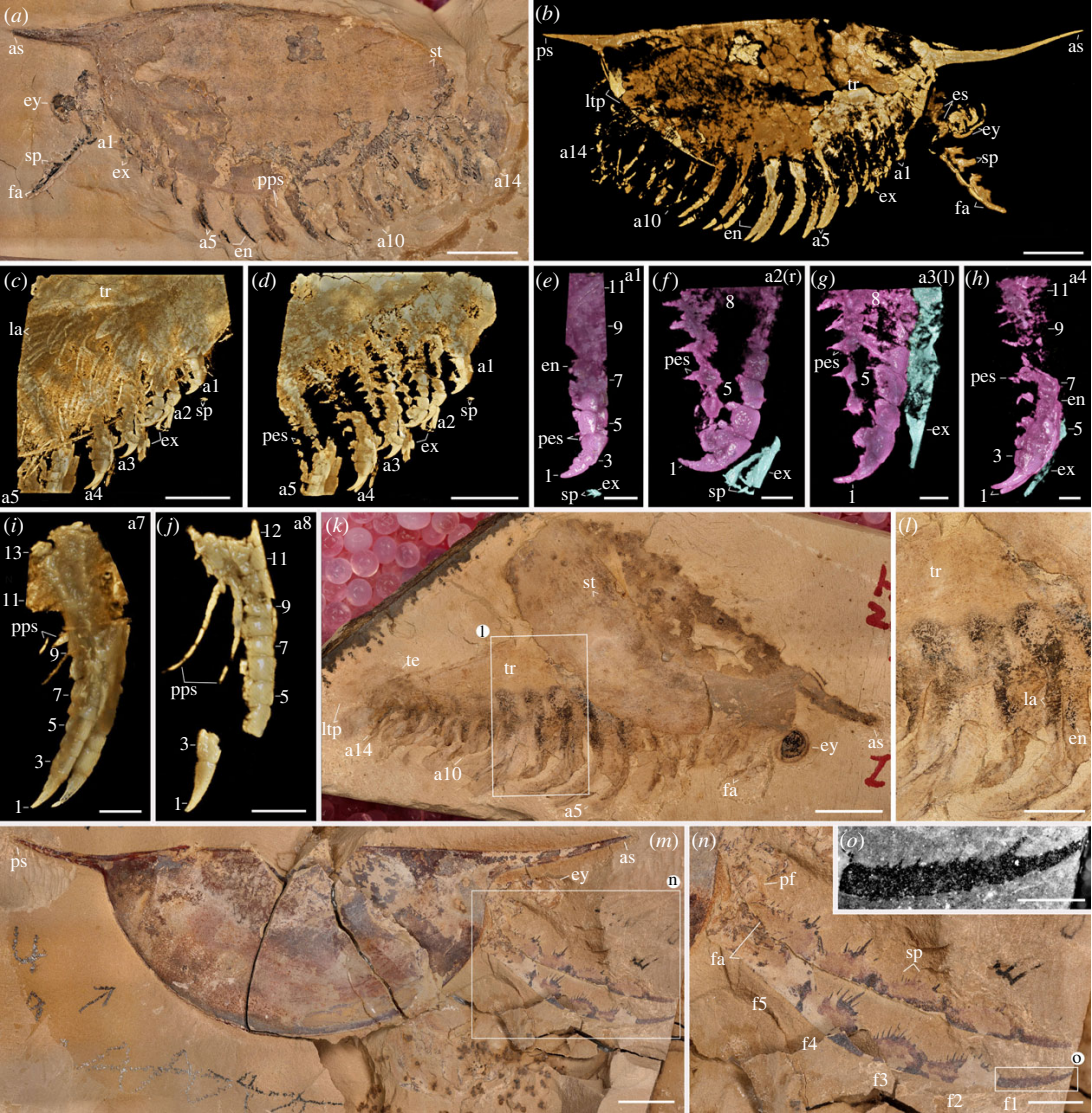
Trunk and appendage morphology of *Isoxys curvirostratus* from the early Cambrian (Stage 3) Chengjiang. (*a–j*) YKLP 16260. (*a*) *Isoxys curvirostratus* photographed under reflected light. (*b*) Tomographic model. (*c*) Anterior trunk region. (*d*) Anterior trunk region with carapace digitally removed. (*e–h*) Isolated first to fourth trunk appendage pair showing morphology of endopod (purple) and exopod (blue) separately. (*i*,*j*) Seventh to eighth appendage pair separately. (*k*,*l*) YKLP 16261a. (*k*) *Isoxys curvirostratus*, showing partially disarticulated carapace and unarthrodized trunk. (*l*) Detail of (*k*). (*m–o*) YKLP 16266. (*m*) *Isoxys* cf. *curvirostratus* with well-preserved frontal appendages. (*n*) Detail of frontal appendages. (*o*) Elongate terminal subchela of (*n*). a*n*, the *n*th ventral appendage; as, anterior spine; en, endopod; es, eye stalk; ex, exopod; ey, eye; fa, frontal appendage; f*n*, podomeres of the frontal appendage from the distal to proximal; la, lamellae of exopod; ltp, lateral processes of telson; pes, paired spines on each endite; pf, proximal part of the frontal appendage; pps, paired posterior spines on endopod; ps, posterior cardinal spine of carapace; sp, spines; st, striated ornament; te, telson; tr, trunk. Numbers indicate endopod podomeres. Scale bars: (*a*,*b*,*k*,*m*), 5 mm; (*c*,*d*), 2.5 mm; (*e*,*i*,*j*,*o*), 1 mm; (*f–h*), 0.5 mm.

**Figure 2.**
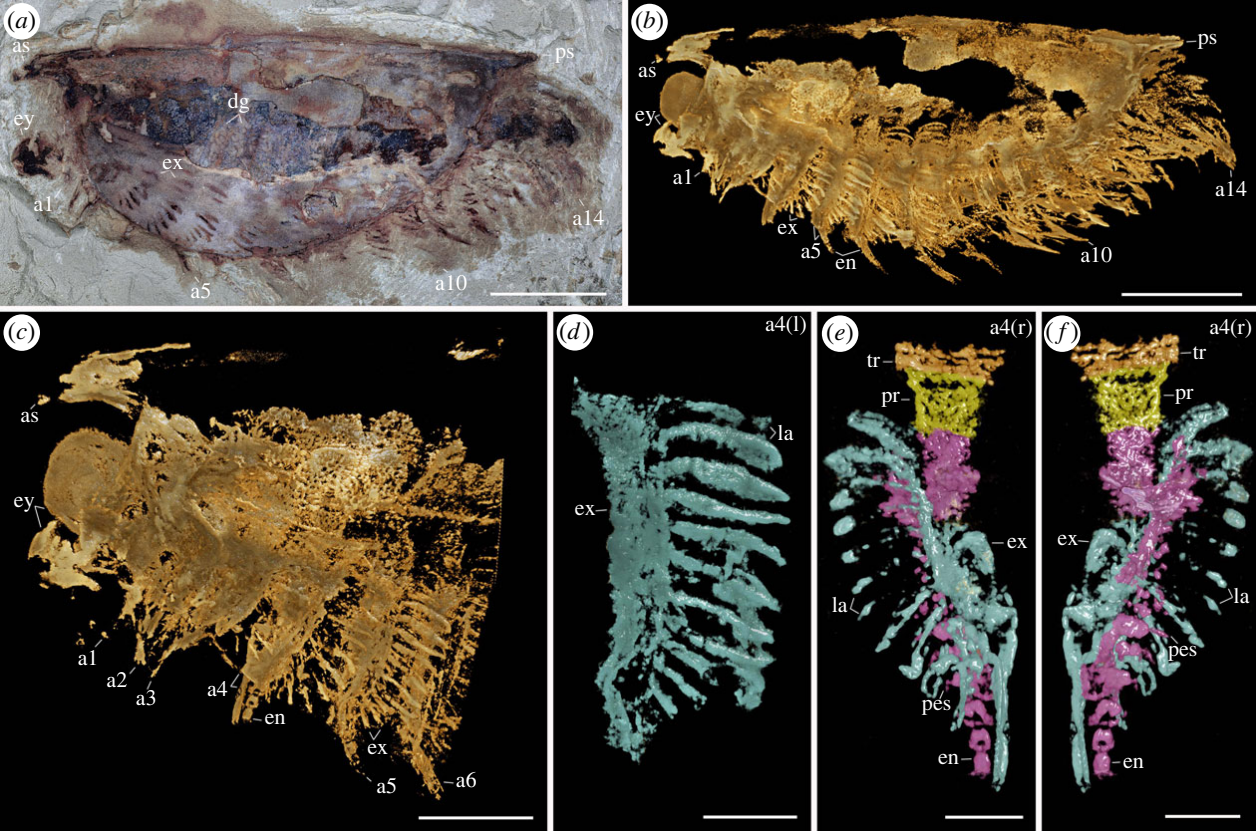
Appendicular morphology of *Isoxys* sp. from the early Cambrian (Stage 3) Chengjiang in South China. (*a*) CFM 00047, complete specimen photographed under reflected light. (*b*) Tomographic model of complete specimen in lateral view. (*c*) Tomographic model showing magnification of anterior body in lateral view with well-preserved appendages. (*d*) Tomographic model of isolated exopod from left fourth appendage in lateral view showing elongate shaft and paddle-shaped lamellae. (*e*) Tomographic model of exopod from right fourth appendage pair in lateral view showing protopod (yellow), endopod (purple) and exopod (blue). (*f*) Tomographic model of exopod from right fourth appendage pair in lateral view, rotated 180°. a*n*, the *n*th ventral appendage; as, anterior spine; dg, digestive gland; ey, eye; la, lamellae of exopod; pes, paired spines on each endite; pr, protopod; ps, posterior cardinal spine of carapace. Scale bars: (*a*,*b*) 5 mm; (*c*) 2.5 mm; (*d–f*) 1 mm.

As typically observed in *Isoxys* fossils [21–26], the studied specimens (figures 1 and 2; and electronic supplementary material, figures S1–S4) are laterally compressed, although the small offset between the appendages from left and right sides in the specimen YKLP 16260 (figure 1*a–d*; electronic supplementary material, figure S2a,b) indicates an oblique-lateral orientation for the ventral appendages. Whereas most of the soft-tissue morphology is encased within the carapace, the stalked eyes, frontal appendages, the distal parts of the ventral trunk appendages and the posterior end of the telson usually extend beyond the carapace margins. In YKLP 16261 (figure 1*k*), the posterior part of the body was partially disarticulated from the carapace, revealing the trunk organization in detail. Specimens YKLP 16260 and CFM 00047 (figures 1*a* and 2*a*) show a strong degree of pyritization, generating a density contrast with the rock matrix that facilitates micro-CT imaging of exceptionally preserved structures (figures 1*b–j* and 2*b–f*).

### (b) Morphological description

We investigated new material of *I. curvirostratus* [23] (figure 1*a*; electronic supplementary material, figures S1–S4), as well as previously published material of *Isoxys* sp. [25] (figure 2) from Chengjiang. *Isoxys* species share fundamental aspects of the overall morphology, including the presence of an extensive and medially folded dorsal carapace with a semicircular smooth margin, anterior and posterior cardinal spines, prominent stalked eyes and robust frontal appendages [23–26] (figures 1 and 2). *Isoxys curvirostratus* is distinguished by the presence of a convex dorsal carapace margin, upward bending anterior cardinal spine approximately three times longer than the posterior spine, and the presence of longitudinal striations on the posterior part of the carapace (figure 1; electronic supplementary material, figures S1–S4). *Isoxys* sp. cannot be ascribed to an existing species due to the lack of diagnostic characters such as the frontal appendages or carapace ornamentation [23,24], and thus is treated in open nomenclature following ref. [25]. Carapace length—measured between the bases of anterior and posterior spines—ranges from 24 to 28 mm for *I. curvirostratus*, and 19 mm for *Isoxys* sp. in our studied material (figures 1 and 2). Comparisons with previous reports on Chengjiang *Isoxys* species [23–26] suggest that our material corresponds to adults based on their size.

Soft tissues have been described for both *I. curvirostratus* and *Isoxys* sp. [23–25]. The anterior end of the body bears a pair of prominent stalked eyes with a spherical shape, and which protrude beyond the anterior carapace margin directly below the cardinal spine (figures 1 and 2; electronic supplementary material, figures S1–S4) [26]. The appearance of the eyes in *Isoxys* closely resembles that of the fossilized ocular structures in other Chengjiang euarthropods [2,3,10], consisting of a light outer layer and a dark internal mass, which probably correspond to the visual surface and retinal pigments, respectively. And the dark internal mass is usually the residual preservation lenses and indicates the adaptation of individual organisms to the water environment at different depths (figure 2*b*,*c*; electronic supplementary material, figure S1) [25,26]. A pair of well-developed frontal appendages is also found in close association with the stalked eyes on the anterior end of the body (figure 1; electronic supplementary material, figures S3 and S4). The frontal appendages appear to attach behind the eyes, similar to *Surusicaris* [17], but their precise position within the head and relative to the mouth opening remains uncertain in our material due to the coverage by the carapace, as well as in other *Isoxys* from Chengjiang [23–25], Sirius Passet [22], Emu Bay Shale [21] and Burgess Shale [18,25]. Our material of *I. curvirostratus* preserves the morphology of the frontal appendage in greater detail than previously described specimens [2,23]. YKLP 16260 and YKLP 16261 shows that the frontal appendage of *I. curvirostratus* consists of six podomeres that are longer (sag.) than wide (trans.), and follows a distinctive curvature in which the ventral side is facing upwards (figure 1*m*,*n*). The basal podomere has a subtrapezoidal shape and lacks endites, whereas the following four podomeres are robust, subequal in length (sag.) and have distinctly curved ventral margin that bears up to a dozen spinose endites that are longer towards the podomere midline, and shorter towards the margins (figure 1*n*; electronic supplementary material, figure S4b). The sixth podomere is a terminal subchela, of subequal length to the previous podomeres but with a slender outline. The terminal subchela also bears spinose endites that are consistently short and point distally (figure 1*o*). The frontal appendages of *I. curvirostratus* are morphologically distinct from those of *I. auritus*, also known from Chengjiang [2,24], as the latter consists of nine podomeres with a subrectangular outline, with subequal length (sag.), each bearing a single median spinose endite.

The carapace of *Isoxys* covers most of the trunk morphology in all specimens described to date [2,18,21–26]. New material of *I. curvirostratus* with a partially displaced carapace informs the organization of the trunk region (figure 1*k*,*l*; electronic supplementary material, figure S4a). YKLP 16261 demonstrates that the trunk of *I. curvirostratus* lacks any indications of dorsal arthrodization such as well-defined tergites or epidermal segmental boundaries, despite the presence of non-biomineralized structures in the same specimen including the eyes, a complete biramous appendage series and paired telson flaps on the posterior end (figure 1*k*; electronic supplementary material, figure S4a). The quality of preservation of YKLP 16261 featuring delicate structures such as the stalked eyes and appendages indicates that this lack of arthrodization is real, rather than a taphonomic artefact caused by decay. The overall surface appearance of the biramous appendages in the studied specimens of *I. curvirostratus* and *Isoxys* sp. is comparable to those in previous reports [2,23–25]. The appendages consist of relatively simple endopods and exopods with setae, and appear nearly homonomous, except for a gentle increase in size from the anterior end to the middle of the body, and then decrease in size from the middle to the posterior end (figures 1*a*,*l* and 2*a*; electronic supplementary material, figure S3). Both *I. curvirostratus* [23] and *Isoxys* sp. feature 14 pairs of ventral biramous appendages (figures 1*a*,*l* and 2*a*,*b*), which distinguish them from the 11 pairs described for the adults of *I. auritus* [24].

**Figure 3.**
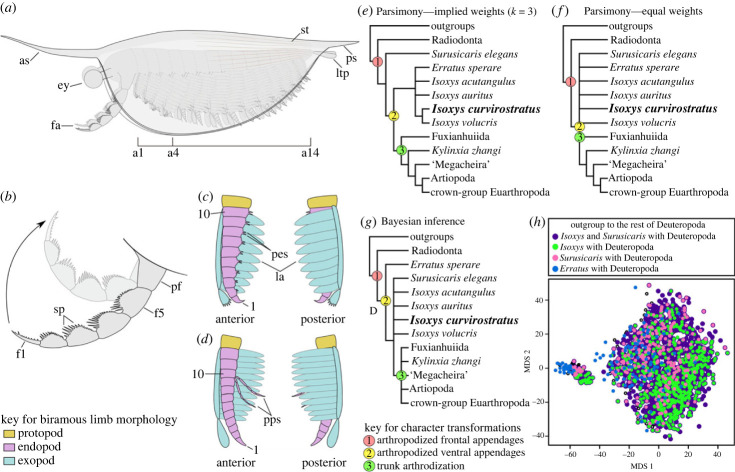
Morphological reconstruction and phylogenetic position of *Isoxys*. (*a*) Lateral view. (*b*) Detail of the frontal appendage. (*c*) Second trunk appendage showing the morphology of the anterior batch of biramous appendages (a1–a4) viewed from anterior (left panel) and posterior (right panel) of the animal. (*d*) Seventh trunk appendage showing the morphology of the posterior batch of biramous appendages (a5–a14) viewed from anterior (left panel) and posterior (right panel) of the animal. (*e*) Simplified strict consensus of maximum parsimony under implied weights *k* = 3 (36 MPTs, 260 steps, CI: 0.650, RI: 0.878). (*f*) Simplified strict consensus of maximum parsimony under equal weights (96 MPTs, 279 steps, CI: 0.606, RI: 0.852). (*g*) Majority rule consensus tree retrieved with Bayesian inference. (*h*) Treespace analysis comparing the distribution of topologies favouring isoxyids versus *Erratus* as the outgroup to other deuteropods. See electronic supplementary material, figures S5 and S6 for detailed results and support values. a*n*, the *n*th ventral appendage; as, anterior spine; ey, eye; fa, frontal appendage; f*n*, podomeres of the frontal appendage from the distal to proximal; la, lamellae of exopod; ltp, lateral processes of telson; pes, paired spines on each endite; pf, proximal part of the frontal appendage; pps, paired posterior spines on endopod; ps, posterior cardinal spine of carapace; sp, spines; st, striated ornament. Numbers indicate endopod podomeres.

Micro-CT imaging and three-dimensional rendering techniques reveal exceptional details of the pyritized limb morphology in Chengjiang fossil euarthropods that are not accessible through conventional light photography [8,27–29]. In *I. curvirostratus*, all the ventral appendages have a biramous construction, with the first to fourth appendage pairs being shorter and morphologically distinct from the subsequent ones (figure 1*b–d*; electronic supplementary material, figure S3c,d). The endopods have well-defined segmental boundaries expressed as regularly spaced transverse grooves along the proximodistal appendage axis. The fact that the grooves are consistent in their position within and between appendages indicates that they are not fractures in the fossil, nor a result from incomplete pyritization. The number and morphology of the endopod podomeres vary between different appendages. The shorter first to fourth biramous appendage pairs have endopods composed of at least 10 robust podomeres, each with a subtrapezoidal outline, and a strongly curved terminal subchela (figures 1*e–h* and 3*c*). Each subterminal podomere carries a pair of medially located endites expressed as strong triangular spines along the ventral margin of the main limb axis (figure 1*f–h*). The exopods of the first to fourth appendage pairs in YKLP 16260 include a slender shaft that is as long as their corresponding endopod (figure 1*f–h*), and bear several thick paddle-shaped lamellae perpendicular to the main limb axis (figure 1*c*; electronic supplementary material, figure S3c,d). The exopod shaft also bears a terminal paddle-shaped lamella with marginal spines on its distal end (figure 1*e*,*f*). The anterior position of the first to fourth post-raptorial appendage pairs and their distinctive morphological specialization suggest that they belong to a functional six-segmented anterior cephalic region, which also includes the segments bearing the stalked eyes and the raptorial frontal appendages. The fifth to fourteenth pairs of biramous appendages also show the preservation of fine morphological details, including endopods with approximately a dozen well-defined transverse podomere boundaries, and which taper in width distally into a terminal subchela, although the latter is more elongate and less curved compared to that of the anterior limbs (figure 1*b*,*i*,*j*; electronic supplementary material, figures S2a and S3b). It is likely that the full podomere count is higher, but details of the proximal portion of the appendages cannot be fully resolved. Unlike the cephalic limbs, the fifth to fourteenth pairs of biramous appendages lack endites on each podomere, but feature a pair of elongate delicate multiarticulated spines at the level of the tenth podomere (figure 1*i*,*j*; electronic supplementary material, figure S1a). The proximal organization of the trunk exopods in *I. curvirostratus* could not be resolved from the studied material, but the paddle-shaped lamellae are visible on the surface of specimens with well-preserved limbs such as YKLP 16260 (figure 1*c*).

The limb morphology of *Isoxys* sp. (figure 2) complements the findings from *I. curvirostratus* to produce a comprehensive understanding of the appendicular organization of *Isoxys*. CFM 00047 features three (rather than four) pairs of smaller biramous appendages, and trunk endopods with more than a dozen podomeres*.* However, *Isoxys* sp. demonstrates that the proximal portion of the biramous appendages consists of an undifferentiated subtrapezoidal protopod (figure 2*e*,*f*). *Isoxys* sp. also shows the complete exopod organization in greater detail, consisting of an elongate shaft that bears at least a dozen thick and paddle-shaped lamellae that attach perpendicular relative to the main exopod axis (figure 2*d*,*f*). The paddle-shaped lamellae imbricate with each other, and lack the fringe of short marginal setae observed in the morphologically similar exopod of Cambrian artiopods (see [28]). Distally, the exopod shaft bears a single paddle-shaped lamella (figure 2*d–f*), as also observed in *I. curvirostratus* (figure 1*e*). CFM 00047 demonstrates that the imbrication of the paddle-shaped lamellae in the exopod can produce an appearance akin to cuticular folds on the surface of the fossils (figure 1*l*) [14,17], which has led to previous misinterpretations of the biramous appendage structure.

## 3. Discussion

### (a) Appendage differentiation in *Isoxys*

New material and the use of micro-CT imaging and three-dimensional rendering techniques to detect the iron-enriched non-biomineralized morphology in pyritized Chengjiang fossils reveal new details of the anatomical organization in *Isoxys* (figure 3*a–d*), with direct implications for its palaeoecological and evolutionary significance. The robust frontal appendages in *I. curvirostratus* are well suited for a raptorial grasping function following an upwards stroke in front of the carapace (figure 3*b*). Combined with the presence of prominent anterior-facing stalked eyes indicates that this euarthropod was an active visual predator in the water column [18,21,23,25,30], possibly up to a depth of 140 m based on morphological estimates [26]. The presence of a short unarthrodized trunk concealed within the carapace suggests that this body region was not efficient for swimming on its own, as it lacks the rigid muscle attachment sites needed for propulsion as observed in other Cambrian forms with cylindrical abdominal sclerite [4,6,27]. Instead, swimming in *Isoxys* was most likely achieved by the rhythmic movement of the biramous appendages powered by the paddle-shaped lamellae on the exopods [25,30] (figure 3). The arthropodized trunk endopods would allow walking on the benthic substrate using the tips of the legs. Whereas the raptorial frontal appendages are well equipped for prey capture thanks to the substantial spinose armature observed in *I. curvirostratus* (figure 1*m–o*), the following biramous appendages indicate a further degree of functional differentiation. We demonstrate that the four anteriormost biramous appendages of *I. curvirostratus* are not only shorter, but that their endopods bear robust paired endites and a strongly curved terminal subchela (*contra* [2,17,18,21,23,25]) (figure 1*e–h*). The integration of the four biramous appendage pairs into a functionally specialized head region suggest that they were used for processing soft-bodied food items drawn into the anterior space within the carapace—once grasped by the frontal appendages—before consumption. By contrast, the undifferentiated protopod and absence of spinose endites on the trunk biramous appendage pairs as observed in *Isoxys* sp. suggest that they were not used for feeding, but instead were exclusively used for locomotion, particularly for active swimming and vertical migration in the water column [30] or walking on the seafloor. The paired slender and multiarticulated spines on the tenth podomere of the trunk endopods are too delicate for food processing; we hypothesize they might have a sensorial function or provide some mechanical support during benthic locomotion. Similar paired and elongate spines have also been recently described in the fuxianuiid *Alacaris*? sp. from the Guanshan biota [31]. In *Alacaris*? sp., the paired spines are also located on the proximal half of the endopod, but in the vicinity of robust protopod gnathobases, which suggest that these delicate spines were not involved in food processing but might suggest their sensorial function. The lack of adaptations for feeding on the trunk biramous appendage pairs of *Isoxys* also argues against a scavenging or detrivorous diet, as these strategies generally require a dense proximal enditic armature that forms a median food groove for processing organic matter [8,32,33]. These findings indicate that the biramous appendages of *Isoxys* possessed a higher degree of heteronomy and functional differentiation than previously considered [1,18,21,23], and reveal an unexpected complexity in the feeding ecology of pelagic predators in early marine ecosystems [25,30].

### (b) Implications for early euarthropod evolution

**Figure 4.**
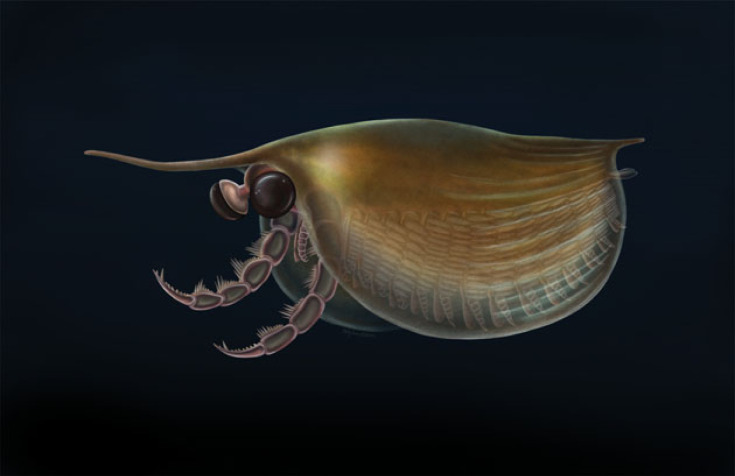
Artistic reconstruction of *Isoxys curvirostratus* from the early Cambrian (Stage 3) Chengjiang biota in South China. Artwork by Holly Sullivan.

*Isoxys curvirostratus* uniquely combines the presence of fully arthropodized biramous appendages, a morphologically and functionally specialized anterior cephalic region, and lack of trunk arthrodization (figures 3 and 4), all of which are critical characters for reconstructing early euarthropod evolution [1,2,5,6]. Notably, *I. curvirostratus* and *Isoxys* sp. demonstrate that all the biramous appendages share similarities with those of deuteropods in a more crown-wards phylogenetic position [5]. The multi-podomerous endopods of *Isoxys* are comparable to those of Cambrian bivalved euarthropods [7,24] and fuxianhuiids [3,31,33], suggesting that they could reflect the ancestral organization of the earliest arthropodized limbs. Similarly, the slender exopod shaft with paddle-shaped lamellae has recently been recognized in a number of Cambrian artiopods, and suggested as potentially symplesiomorphic for that clade [28]. The Burgess Shale *Surusicaris* also has three anteriormost differentiated ventral appendages [17], indicating that the presence of a multi-segmented head region is widespread among isoxyids. Critically, an isoxyid-like cephalization pattern has been recently recognized in the early diverging stem-group euarthropod *Kylinxia* from Chengjiang [2], which strikingly also possesses upwards-facing raptorial frontal appendages and four pairs of smaller biramous appendages in the head. *Isoxys* and *Kylinxia* also share the presence of paired telson flaps, although other aspects of their body morphology differ substantially, such as the presence of trunk tergites and absence of a carapace in the latter. Indeed, the lack of trunk arthrodization in *Isoxys* is comparable with the absence of epidermal dorsal segmentation observed in radiodonts [12,15,34], which suggests that *Isoxys* embodies an earlier step than the fully arthrodized *Kylinxia* in the evolutionary history of the euarthropod body plan. This interpretation is further supported by the results of our phylogenetic analyses (see below), in which *Kylinxia* is consistently recovered in a more crown-wards position relative to isoxyids (figure 3) (*contra* [2]). The arthropodized appendages of *Isoxys* also differ from those of *E. sperare* [20] in several important aspects. First, *Isoxys* appendages appear to be more heavily sclerotized based on the well-defined podomere boundaries and the robust spinosity of the frontal appendages (figure 1), whereas the endopods of *Erratus* are only weakly sclerotized as indicated by delicate transverse podomere boundaries [20]. Second, the cephalic biramous appendages of *Isoxys* feature robust triangular paired endites, terminate in a strong curved subchela, and show a notable change in their size and morphology along the anterio posterior body axis, whereas all the biramous appendages of *Erratus* are conical, without endites or other feeding adaptations, and maintain a subequal length throughout the body. In this context, *Isoxys* limbs reflect a more advanced degree of morphological and functional specialization and regionalization relative to *E. sperare* [20]. Although previous studies have suggested that the body of isoxyids was at least weakly sclerotized [7,18], our data conclusively demonstrates that the trunk of *I. curvirostratus* was completely unarthrodized, and allows us to explore its implications for the origin of the archetypical euarthropod body plan in greater detail.

The results of phylogenetic analyses using maximum parsimony and Bayesian inference (figure 3*e–g*; electronic supplementary material, figures S5 and S6) to explore the evolutionary implications of our new morphological data provide support for the hypothesis that isoxyids generally, and genus *Isoxys* specifically, may be the outgroup all other Deuteropoda, rather than *Erratus* or *Kylinxia* (*contra* [2,20], respectively). The strict consensus of the maximum-parsimony analysis with implied weights (figure 3*e*; electronic supplementary material, figure S5a) resolves *Surusicaris* as the earliest branching taxon within Deuteropoda, and *Isoxys* species as part of a weakly supported clade that also includes *Erratus*. In this analysis, *Kylinxia* is resolved in a more crown-wards position within Deuteropoda. Under equal weights (figure 3*f*; electronic supplementary material, figure S5b), the base of Deuteropoda is collapsed forming a polytomy that comprises all isoxyids and *Erratus*. By contrast, Bayesian inference recovers *Erratus* as the earliest branching member of Deuteropoda (as in [20]), and isoxyds in a basal polytomy relative to other deuteropods (figure 3*g*; electronic supplementary material, figure S5c).

We used treespace analysis [34] to visualize the topologies retrieved by our phylogenetic reconstruction (*n* = 2108 trees total), and the degree of uncertainty at this node (electronic supplementary material, figure S6), information that is omitted when viewing a consensus tree alone. Indeed, 87% (*n* = 1825 trees) of retrieved topologies support isoxyids (i.e. genus *Isoxys* plus *Surusicaris*) as the outgroup to Deuteropoda, to the exclusion of *Erratus*. A further 28% (*n* = 590 trees) of topologies supports *Isoxys* as a sole outgroup to deuteropods, while 11% (*n* = 231 trees) of the trees have *Surusicaris* alone in this position. Topologies indicating *Erratus* alone is the outgroup of Deuteropoda represent only 5% (*n* = 109 trees). In the treespace itself, topologies form distinct clusters depending on whether they were analysed using parsimony or Bayesian methods (electronic supplementary material, figure S6b), but both clusters include trees with each possible bipartition (electronic supplementary material, figure S6a). The topologies supporting *Erratus* are more abundant on the side of the *x*-axis that contains the parsimony cluster, while isoxyids are everywhere (electronic supplementary material, figure S6a) and probably do not represent a local optimum. Ultimately, our results provide evidence that isoxyids are viable candidates as the sister group of Deuteropoda, and a single origin for dorsal trunk arthrodization in Deuteropoda is the most likely scenario. Our findings also contrast with the recent interpretation that isoxyids are occupy a crown-wards position relative to *Kylinxia*, which would imply that the lack of trunk arthrodization in these bivalved euarthropods is the result of secondary loss (*sensu* the topology of [2]). Although the loss of substantial body sclerotization is well known in phylogenetically derived extant euarthropods (e.g. opisthothele aranaeids, pentastomids), our results strongly support the hypothesis that the unarthrodized body of *Isoxys* reflects an ancestral condition. The reinvigorated understanding of the body organization of *Isoxys* made possible by new fossil material and micro-CT imaging consolidates their key role in the step-wise evolution of the fundamental exoskeletal characters that define crown-group Euarthropoda.

Our results indicate that the precise phylogenetic position of *Isoxys*, *Surusicaris* and *Erratus* is highly sensitive to the type of analysis, but these taxa are consistently recovered as the earliest branching members of Deuteropoda, and thus inform the early macroevolution of archetypical euarthropod characters. Regardless of the topology, all analyses recover a single evolutionary event for the origin of arthropodized frontal appendages in the node including Radiodonta + Deuteropoda. Both implied weights parsimony and Bayesian inference indicate the arthropodization of the ventral appendages precedes the arthrodization of the trunk region, whereas equal weights parsimony is ambiguous due to a lack of resolution. Although this hypothesis has been put forward [20], the dorsal trunk is not clearly observable in previously published specimens of *Isoxys* [21–26], *Erratus* [20] or *Surusicaris* [17], and thus the availability of new material of *I. curvirostratus* (figure 1*k*; electronic supplementary material, figure S4a) allows us to strengthen this interpretation. More broadly, this result is consistent with the view that the combination of characters that typify the body plan of crown-group Euarthropoda did not evolve simultaneously, as might erroneously appear from the diversity of stem-group euarthropods in the fossil record, but rather sequentially among the earliest branching members of Deuteropoda [5].

## 4. Material and methods

### (a) Material

All studied specimens were collected from the mudstones in the Cambrian Stage 3 Yu'anshan Member of the Chiungchussu Formation. The specimens YKLP 16260–16264 were collected from the Haikou area of Kunming, China, and are housed at the Yunnan Key Laboratory for Palaeobiology, Yunnan University. Specimen CFM 00047 was collected from the Xiaolantian section in Chengjiang, and is housed at the Chengjiang Fossil Museum, Yuxi.

### (b) Fossil imaging

Fossil specimens were photographed with a Keyence VHX 6000 and Leica M205AF stereomicroscope. In order to observe the structures buried within the rock matrix and to produce three-dimensional models of the preserved morphology, micro-CT imaging and three-dimensional computer rendering techniques were applied. The best outcome is from specimens YKLP 16260 and CFM 00047. Specimen YKLP 16260 was first scanned with a GE Phoenix Nanotom cone beam scanner at the Bavarian State Collection of Zoology, Bavarian Natural History Collections, München, Germany, to detect the signal/noise ratio, and then with a Zeiss Xradia 520 Versa X-ray microscope at Yunnan Key Laboratory for Palaeobiology to obtain images with higher resolutions. For the Xradia 520 Versa scanning, the energy and the resolution were set at 60 kv/5w and 15.25 µm for slab a, and at 60 kv/5w and 15.89 µm, 50 kv/4w and 8.48 µm for slab b (scanned twice), to obtain a higher resolution of the anterior part of the body. Specimen CFM 0047 was scanned with a Zeiss Xradia 520 Versa X-ray microscope with the above two parameters set at 70 kv/6w and 5.41 µm for overview scanning, and at 60 kv/5w and 11.6 µm for small-field, detailed scanning.

### (c) Phylogenetic analysis

To assess the phylogenetic position of *Isoxys*, we analysed an updated version of the published morphological matrix of Pates *et al*. [35]. We coded *I. curvirostratus* according to new anatomical data herein, and added *I. acutangulus*, *I. auritus*, *I. volucris*, *Surusicaris elegans* and *E. sperare* from previous literature*.* Therefore, the matrix comprised 63 taxa and 135 discrete characters. Details of all characters including character descriptions and scorings may be downloaded from MorphoBank [36] (https://morphobank.org, doi:10.7934/P4286).

We analysed the morphological dataset in MrBayes v.3.2.7 [35], implementing the Mk model of character evolution [37] with gamma distributed among-character rate variation for four runs of four chains and 10 million generations, with 25% burnin. Convergence was assessed based on standard deviations of split frequencies less than 0.01, reaching effective sample size greater than 200 for every parameter, and by comparing posterior distributions in Tracer v.1.7.1 [38]. We also analysed the morphological matrix using maximum parsimony in TNT v.1.5 [39] using both equal weights and implied weights (*k* = 3). For both weighting schemes, we required the shortest tree to be retrieved 100 times, and used tree bisection–reconnection to swap one branch at a time on the trees in memory.

We further interrogated support for alternative phylogenetic positions of isoxyids using treespace visualization [34,40,41]. As described in [34], which originated the implementation, this method calculates pairwise unweighted Robinson–Foulds (RF) distances for the total set of unrooted trees (Bayesian and maximum parsimony) using *phangorn* v.2.5.5 [42,43] and visualizes the RF distances using classical multidimensional scaling in *ape* v.5.3 [44]. See [34] for additional details of the method.

## Data accessibility

All data analysed in this paper are available as part of the article, or within the electronic supplementary material (figures S1–S7 or Information) [45]. Original tomographic datasets are available on Dryad Digital Repository (doi:10.5061/dryad.f4qrfj718) as greyscale TIFF images, and are freely accessible for visualization. Phylogenetic results are available at the same Dryad link, while the detailed morphological matrix is available at MorphoBank (doi:10.7934/P4286).
